# Common Sickness

**Published:** 1869-03

**Authors:** 


### COMMON SICKNESS.

When one man gets mad at the stupidity of another, he calls him a goat, a goose or an ass; but these much abused animals are respectable as to their acquirements in the judicious treatment of themselves when ailing, in comparison with the masses of humanity; in comparison, indeed, with many persons of superior intelligence and culture; these, in common with other animals do three things when suffering: First, they court quiet; second, practice abstinence as to food; third, seek warmth. There is reason to believe that if these three things were promptly and judiciously attended to, that three-fourths of all human ailments would be more speedily alleviated, and would be ultimately cured with a greater certainty than by the aid of all the medicines ever known; not that medicine in the hands of an intelligent physician is undervalued, but that natural remedies are overlooked. We call quiet, abstinence and warmth "natural remedies," because our instincts promptly indicate them when disease invades the system. When we do not feel well, we become indisposed to move about, we want to lie down, we do not even care to make the exertion to talk; very generally we feel chilly and hover around the fire, and almost always we very soon begin to loathe the very thought of food. And yet it is safe to say that four persons out of five, as soon as they begin to feel "out of sorts," as it is expressed, bethink themselves of what they shall take, and too many "take a drink," that is, begin to swill brandy, rum, gin, whiskey, or more vulgar beer. Why, a "calf" would n't do such a thing, but man, with all his boasted intelligence, does, and thus proves himself the greatest calf or goose or goat of them all.

